# Timing of systemic therapy relative to stereotactic radiosurgery for brain metastases: a real-world cohort study

**DOI:** 10.1007/s11060-026-05857-0

**Published:** 2026-11-03

**Authors:** Mijal Ptascinsky, Antoni Skripai, Yair Hillman, Marc Wygoda, Aron Popovtzer, Tal Falick Michaeli

**Affiliations:** 1 Department of Radiation Oncology, Sharett Institute of Oncology, Hebrew University Medical Center, Jerusalem, Israel; 2 Department of Military Medicine and “Tzamert”, Faculty of Medicine, Hebrew University of Jerusalem, Jerusalem, 9112002 Israel; 3 Faculty of Medicine, Department of Developmental Biology and Cancer Research, Institute for Medical Research Israel-Canada, Hebrew University of Jerusalem, Jerusalem, Israel

**Keywords:** Stereotactic radiosurgery (SRS), Brain metastases, Immunotherapy, Targeted therapy, Chemotherapy, Treatment timing

## Abstract

**Purpose:**

Combining stereotactic radiosurgery (SRS) with systemic therapy is increasingly common for brain metastases, yet real-world evidence on immunotherapy timing relative to SRS remains limited. We evaluated the association of immunotherapy and its timing with survival, intracranial control and toxicity, with exploratory analyses of chemotherapy and targeted therapy.

**Methods:**

We retrospectively analyzed 155 patients with 556 brain metastases treated with SRS at a single tertiary center (January 2021–February 2025). Patient-level associations of systemic therapy, overall and by timing (before, concurrent with or after SRS), with overall survival, 1-year local, distant intracranial and systemic progression, toxicity and radionecrosis were assessed using Kaplan–Meier, Cox and logistic regression analyses, with false discovery rate correction for immunotherapy timing.

**Results:**

Median follow-up was 18.8 months. Immunotherapy (*n* = 88) was not associated with overall survival (adjusted HR 0.79, 95% CI 0.46–1.35), intracranial progression or toxicity, and no timing window remained significant after correction (all q ≥ 0.53). In exploratory analyses, targeted therapy was associated with longer survival (adjusted HR 0.45, 95% CI 0.26–0.79) and with more frequent radionecrosis (13.2% vs. 3.5%; *p* = 0.034), the latter not robust to sensitivity analysis. A higher number of treated metastases was independently associated with local (aOR 1.15, 95% CI 1.02–1.30) and distant intracranial progression (aOR 1.45, 95% CI 1.24–1.71).

**Conclusion:**

In this single-institution real-world cohort, immunotherapy and its timing relative to SRS were not associated with survival, intracranial control or toxicity. Exploratory associations of targeted therapy with survival and radionecrosis are hypothesis-generating and warrant prospective, histology-specific validation.

**Supplementary Information:**

The online version contains supplementary material available at https://doi.org/10.1007/s11060-026-05857-0.

## Introduction

Brain metastases occur in 20–50% of patients with solid malignancies, particularly from lung, breast, melanoma, and renal cell carcinoma [1–3], with median overall survival ranging from 7 to 12 months depending on primary tumor type [4, 5].

Stereotactic radiosurgery (SRS) has largely replaced whole-brain radiotherapy (WBRT) for brain metastases [1, 3, 6], offering improved local control, reduced toxicity, and preserved cognitive function and quality of life [1–3, 7–9], although overall survival is comparable to WBRT and distant brain recurrence may be higher [2, 3, 9].

Combining SRS with immune checkpoint inhibitors (ICIs) may improve overall and progression-free survival compared with either therapy alone, albeit with a potential increase in adverse events [10–14]. Shorter intervals between SRS and ICIs, regardless of sequence, have been associated with improved local control and survival without increased radionecrosis [15–17], including in patients with multiple or symptomatic lesions [18], and administration within four weeks of SRS has been suggested to offer the most favorable balance of efficacy and toxicity [3, 6, 11–13, 19, 20].

Hypofractionation may improve local control [3], and low radiation doses can stimulate T-cell activity [21]. Chemotherapy may reduce new brain metastases but can increase radionecrosis risk [19], and total intracranial tumor volume is a key determinant of survival and need for salvage therapy [3, 11, 12, 21]. Beyond immunotherapy and cytotoxic chemotherapy, targeted agents are increasingly central to the systemic management of tumor types prone to brain metastasis, and their variable intracranial activity and potential radiation effects raise similar questions regarding sequencing with SRS [13, 19].

Real-world data on how systemic therapy and its timing interact with SRS remain limited. This study aimed to compare outcomes after SRS in patients who did and did not receive immunotherapy, and to evaluate the association between immunotherapy timing and outcomes. The associations of chemotherapy, targeted therapy and other treatment-related factors with SRS outcomes were examined in exploratory analyses.

## Materials and methods

This retrospective cohort study included patients with brain metastases from extracranial primary tumors whose first SRS course was delivered at Hadassah Ein Kerem Medical Center between 1 January 2021 and 28 February 2025; the data cut-off was 31 July 2025.

### Inclusion and exclusion criteria

Patients were excluded if they had a primary brain tumor, received their first SRS course before 2021 or at another institution, or had insufficient follow-up, defined as no post-SRS clinical or imaging documentation in our records or loss to follow-up within 2 months of SRS without documented death; patients who died during this period were retained. Of 459 patients screened, 304 were excluded (primary brain tumor, *n* = 292; insufficient follow-up, *n* = 10; first SRS course before 2021, *n* = 2), leaving 155 patients: 88 who received immunotherapy (with or without other systemic therapy) and 67 who did not.

### SRS treatment planning and delivery

Patients were immobilized with a personalized thermoplastic mask. CT was fused with MRI for dose calculation and target delineation, and conformal plans (4–5 non-coplanar dynamic conformal arcs, or VMAT for complex or organ-at-risk-adjacent targets) were generated in Brainlab Elements v4 and delivered on TrueBeam linear accelerators (Varian) with ExacTrac (Brainlab) image guidance.

Dose was prescribed to the 80% isodose line encompassing the target (maximum dose 125% of the prescription dose). Single-fraction SRS or fractionated stereotactic radiotherapy (FSRT), delivered on consecutive days, was selected according to lesion size and proximity to critical structures, as previously described for large metastases at our institution [22].

Re-irradiation was defined as salvage irradiation of a previously treated metastasis after in-field progression, distinct from upfront FSRT (a planned multi-fraction course) and from SRS to new metastases (recorded as a separate course). Whole-brain radiotherapy (WBRT) was not systematically recorded.

### Clinical variables and definition of outcomes

Data were collected from medical records and anonymized. Variables included age, sex, smoking, Charlson comorbidity index, Eastern Cooperative Oncology Group (ECOG) performance status at SRS, primary tumor site and synchronous or metachronous presentation; systemic therapy and its timing; metastasis number, location and volume (gross tumor volume), with cumulative intracranial tumor volume (CITV) calculated per patient as the sum of all treated metastasis volumes when available for all lesions; and fractionation, prescribed dose and re-irradiation status of each metastasis.

Systemic therapy timing was defined per patient relative to the first SRS course, based on administration dates; subsequent courses did not modify the classification. A therapy was classified as pre-SRS or post-SRS if any dose was given before or after SRS, respectively. For immunotherapy, three further categories were defined: concurrent (an active immunotherapy course on the day of SRS), and within 4 weeks before or after SRS (any dose within the preceding or following 28 days). Categories were not mutually exclusive, and a patient could contribute to several (Fig. S1). For chemotherapy and targeted therapy, only pre- and post-SRS administration was assessed, without a pharmacokinetic definition of concurrency. Start dates of post-SRS therapies were not recorded. Patients with missing treatment dates (immunotherapy, *n* = 3; chemotherapy, *n* = 1; targeted therapy, *n* = 3) were excluded from the corresponding timing analyses. Targeted therapy comprised kinase inhibitors, monoclonal antibodies and antibody–drug conjugates (Table S1); endocrine agents were recorded as hormonal therapy.

Follow-up was performed according to routine institutional practice without a fixed imaging protocol, relying primarily on brain MRI, supplemented by CT and PET-CT. Patients underwent a median of 3 post-SRS brain imaging studies (IQR 2–5); among 137 patients with at least 3 months of follow-up, the median was 3.4 studies per year (IQR 2.7–4.8). All 9 patients without post-SRS imaging died within 2 months of SRS. MRI sequence details were not recorded.

Local progression was defined as in-field progression of at least one treated metastasis, and distant intracranial progression as at least one new brain metastasis outside the treated fields, based on reports by specialist radiologists and the treating oncologists’ documentation. Systemic progression was defined as extracranial progression on CT or PET-CT or as documented by the treating oncologist. Progression was assessed within 12 months of the first SRS course; patients who died without progression were classified as not having progressed, so that death was treated as a competing event. SRS to new metastases was counted as distant intracranial progression.

Toxicity was graded retrospectively according to CTCAE v5.0 from the documented clinical management, recording the highest grade per patient. Radionecrosis was diagnosed primarily on conventional MRI, as reported by the radiologist and/or assessed by the treating radiation oncologist, and graded using the CTCAE term “central nervous system necrosis”; perfusion imaging and histopathology were not routinely performed. Time to radionecrosis was measured from the first SRS course. Corticosteroid use was defined as administration on the day of SRS, with doses converted to dexamethasone equivalents (0.75 mg dexamethasone = 5 mg prednisone); no institutional protocol governed corticosteroid use.

Histological subtype was recorded only for lung primaries; molecular profiling (e.g., EGFR, ALK or BRAF status), Karnofsky Performance Status and the extracranial disease data required for recursive partitioning analysis (RPA) class were not systematically recorded.

### Statistical analysis

All analyses were performed at the patient level, with each patient contributing one observation. Metastasis characteristics were summarized per patient (number of treated metastases, CITV, involvement of each location and FSRT for at least one metastasis), and lesion-level data are reported descriptively only (Table 1, Part B).

Overall survival was measured from the first SRS course to death from any cause, with surviving patients censored at last follow-up. Median follow-up was estimated using the reverse Kaplan–Meier method, and potential follow-up from the first SRS course to the data cut-off.

The comparison of patients who did and did not receive immunotherapy was prespecified as the primary analysis. Immunotherapy timing subgroups were secondary analyses, with p values adjusted using the Benjamini–Hochberg false discovery rate (q values) [23], and chemotherapy, targeted therapy, radiotherapy and location analyses were exploratory. Each exposure, including each timing subgroup, was compared with patients who never received the corresponding therapy (Tables 2, 3 and S2). Baseline characteristics were compared using the chi-square or Fisher’s exact test and the Mann–Whitney U test (Table S3). Overall survival was compared using the Kaplan–Meier method and log-rank test, with hazard ratios (HR) from Cox regression and 12-month estimates with 95% confidence intervals (CI) based on Greenwood’s standard error. One-year progression and toxicity were compared using the chi-square test and radionecrosis using Fisher’s exact test, with odds ratios (OR) where applicable. Multivariable Cox and logistic regression models were adjusted for age, sex, ECOG performance status (0–1 vs. ≥ 2), primary tumor site (lung vs. other) and number of treated metastases, limiting covariates to approximately 10 events per variable. No model was fitted for radionecrosis (12 events); instead, sensitivity analyses restricted targeted therapy exposure to agents started before radionecrosis diagnosis and restricted the cohort to patients with at least 12 months of potential follow-up. Corticosteroid dose was compared by radionecrosis status using the Mann–Whitney U test, and an exploratory sensitivity analysis was restricted to patients with NSCLC. Analyses were performed in SPSS 22.0 (false discovery rate in Microsoft Excel), with two-sided tests and a significance threshold of 0.05.

## Results

### Baseline demographic and clinical characteristics

The cohort included 155 patients with 556 treated metastases (Table 1). Median age was 66 years (range, 16–93), and the lung was the most common primary site (49.7%). Most patients (94.8%) had received systemic therapy before SRS; during follow-up, 88 (56.8%) received immunotherapy, 111 (71.6%) chemotherapy and 68 (43.9%) targeted therapy. Of 556 metastases, 463 (83.3%) were treated with single-fraction SRS, and 21 (3.8%) received salvage re-irradiation.

Median follow-up was 18.8 months (95% CI 14.6–23.0), and median potential follow-up 24.7 months (range, 5.6–43.1); 151 patients (97.4%) had at least 6 months and 130 (83.9%) at least 12 months of potential follow-up. Sixty-six patients (42.6%) died; median overall survival was 22.0 months (95% CI 14.6–29.4), and 1-year overall survival 66.5% (95% CI 58.5–74.5). Within 1 year, 63 patients (40.6%) had local, 72 (46.5%) distant intracranial and 99 (63.9%) systemic progression. Toxicity occurred in 51 of 154 evaluable patients (33.1%; grade 1, *n* = 34; grade 2, *n* = 15; grade 3, *n* = 2; no grade 4–5). Radionecrosis developed in 12 patients (7.7%; grade 2, *n* = 11; grade 3, *n* = 1), all symptomatic, at a median of 10.5 months after SRS (range, 5.6–38.9); among 130 patients with at least 12 months of potential follow-up, it occurred in 11 (8.5%). Corticosteroid dose did not differ by radionecrosis status (median 3 vs. 4 mg; *p* = 0.360).

**Table 1:** Baseline patient, metastasis and treatment characteristics of the study population

PARAMETERS	VALUE
**A. Patient characteristics (** *n* ** = 155)**	
DEMOGRAPHIC	
Age (years)	66 (16–93)
Sex	
Female	77 (49.7%)
Male	78 (50.3%)
Current or former smoker	87 (56.1%)
BASELINE CLINICAL CHARACTERISTICS	
Charlson comorbidity index	9 (6–15)
ECOG performance status	
0–1	90 (58.1%)
≥ 2	53 (34.2%)
Not documented	12 (7.7%)
Corticosteroids on the day of SRS	148 (95.5%)
Dexamethasone-equivalent dose (mg)^a^	4 (2–16)
PRIMARY TUMOR SITE	
Lung	77 (49.7%)
NSCLC - adenocarcinoma	23 (29.9%)
NSCLC - squamous cell	4 (5.2%)
NSCLC- Large cell carcinoma	1 (1.3%)
NSCLC - NOS	39 (50.6%)
SCLC	9 (11.7%)
Not documented	1 (1.3%)
Melanoma	19 (12.3%)
Breast	18 (11.6%)
Gastrointestinal	11 (7.1%)
Gynecological	9 (5.8%)
Kidney	8 (5.2%)
Other ^b^	12 (7.7%)
Not documented	1 (0.6%)
INTRACRANIAL DISEASE	
Number of treated metastases per patient	2 (1–27)
Cumulative intracranial tumor volume (cm^3^)	1.39 (0.02–34.30)
Not documented	9 (5.8%)
Presentation	
Synchronous	53 (34.2%)
Metachronous	102 (65.8%)
Laterality	
Right hemisphere only	41 (26.5%)
Left hemisphere only	41 (26.5%)
Bilateral	68 (43.9%)
Not documented	5 (3.2%)
Leptomeningeal spread	2 (1.3%)
SYSTEMIC TREATMENTS	
Any systemic treatment prior to SRS	147 (94.8%)
Immunotherapy	
Yes	88 (56.8%)
Within 4 weeks prior to SRS ^c^	27 (30.7%)
Within 4 weeks after SRS ^c^	48 (54.5%)
After SRS	63 (71.6%)
No	67 (43.2%)
Chemotherapy ^f^	111 (71.6%)
Targeted therapy ^f^	68 (43.9%)
Hormonal therapy ^f^	5 (3.2%)
B. Metastasis and treatment characteristics (*n* = 556)	
METASTASIS CHARACTERISTICS	
Metastasis volume (cm^3^)	0.36 (0.01–59.23)
Not documented	71 (12.8%)
Laterality	
Right hemisphere	212 (38.1%)
Left hemisphere	227 (40.8%)
Not documented	117 (21.0%)
Anatomical location	
Frontal lobe	134 (24.1%)
Parietal lobe	65 (11.7%)
Occipital lobe	59 (10.6%)
Temporal lobe	46 (8.3%)
Cerebellum	98 (17.6%)
Overlapping lobes ^d^	28 (5.0%)
Other location ^e^	33 (5.9%)
Not documented	93 (16.7%)
RADIATION CHARACTERISTICS	
Fraction scheme (first course)	
Single fraction	463 (83.3%)
Total dose (Gy)	20 (14–27)
2–3 fractions	74 (13.3%)
Total dose (Gy)	27 (18–39)
5–7 fractions	19 (3.4%)
​ Total dose (Gy)	36 (25–48)
Re-irradiation course	21 (3.8%)
≥ 2 re-irradiation courses	2 (0.4%)

Notes: Abbreviations: ECOG = Eastern Cooperative Oncology Group; Gy = Gray; SRS = Stereotactic Radiosurgery; NOS = not otherwise specified; NSCLC = non-small cell lung cancer; SCLC= small cell lung cancer

Continuous variables are presented as median (range). Categorical variables are presented as number of patients (%). Percentages in part A are calculated among all patients (*n* = 155) unless otherwise indicated; percentages for lung histological subtypes are calculated among patients with lung primaries (*n* = 77). Systemic treatment categories are not mutually exclusive, and systemic treatment data were not documented for one patient. Part B reports per metastasis (*n* = 556); prescribed doses refer to the first SRS course, and laterality was not documented for 117 metastases and location for 93

^a^ Among patients receiving corticosteroids (*n* = 148); prednisone doses (*n* = 2) were converted to dexamethasone-equivalent doses

^b^ Bladder (*n* = 6), sarcoma (*n* = 2), Merkel cell carcinoma (*n* = 1), sebaceous carcinoma of the skin (*n* = 1), thyroid (*n* = 1) and pleural mesothelioma (*n* = 1)

^c^ Percentages of patients receiving immunotherapy (*n* = 88); timing windows overlap. All timing categories are shown in Table 2. Timing could not be determined for 3 patients

^d^ Spanning two adjacent lobes: frontal-parietal (*n* = 12), occipital-parietal (*n* = 8), temporal-parietal (*n* = 4), frontal-temporal (*n* = 3) and temporal-occipital (*n* = 1)

^e^ Brainstem (*n* = 11), deep gray matter (*n* = 12), periventricular, intraventricular or pineal (*n* = 4), extra-axial or dural (*n* = 3), insula or cingulate (*n* = 2) and optic chiasm (*n* = 1)

^f^ Not documented for one patient

### Systemic treatments and disease progression

#### Immunotherapy (IO)

**Fig. 1:**
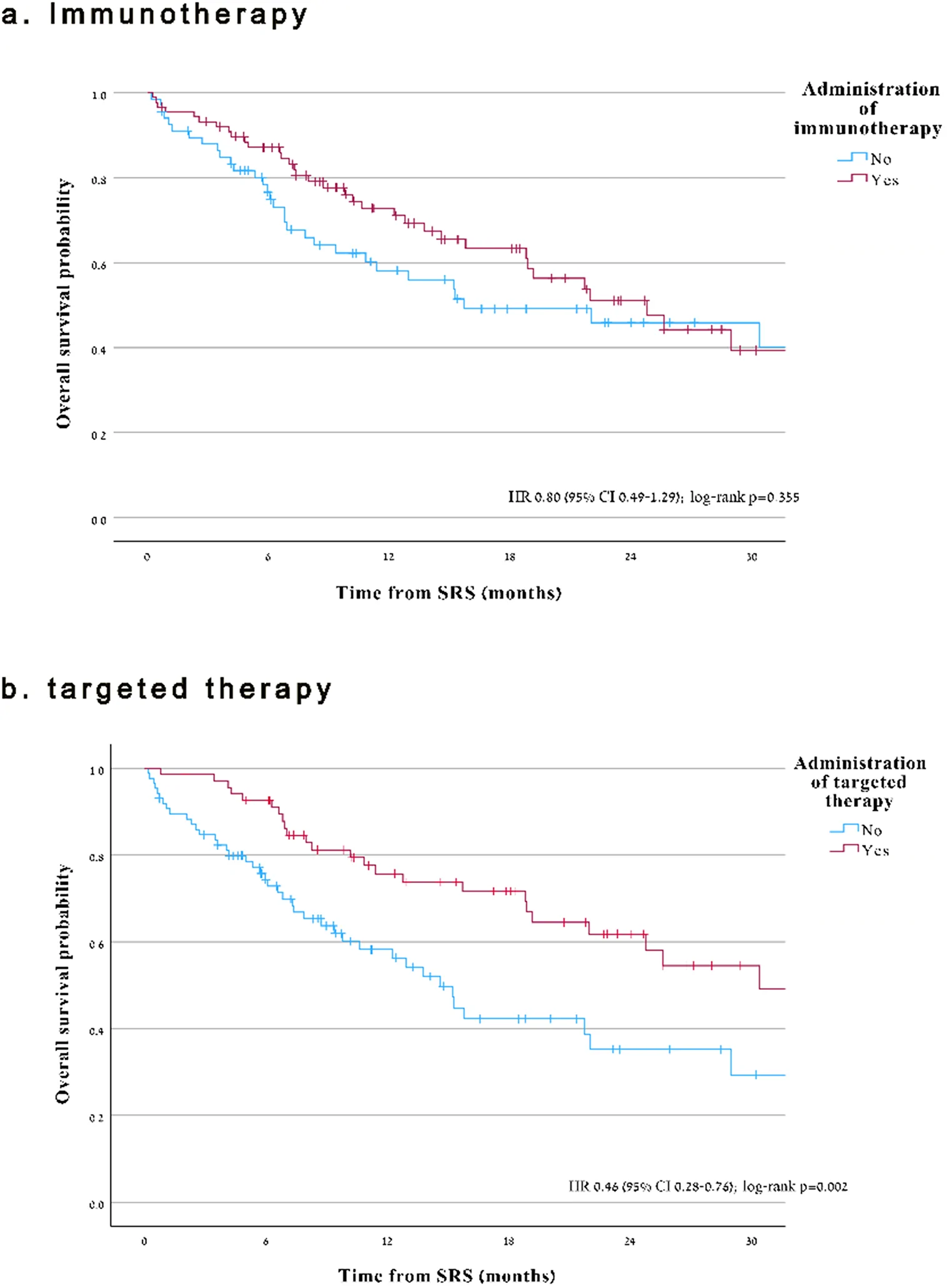
Kaplan–Meier estimates of overall survival by immunotherapy (**a**) and targeted therapy (**b**). Abbreviations: CI = confidence interval; HR = hazard ratio; SRS = stereotactic radiosurgery. Overall survival from SRS in patients who did versus did not receive immunotherapy (**a**) or targeted therapy (**b**) at any time. Tick marks indicate censored patients. HR, unadjusted Cox hazard ratio; p, log-rank test. Targeted therapy analyses are exploratory; post-SRS exposure is susceptible to immortal-time bias, and patients eligible for targeted therapy may differ in prognosis. Numbers at risk at 0, 6, 12, 18, 24 and 30 months: (**a**) immunotherapy 88, 68, 42, 30, 16, 7; no immunotherapy 67, 44, 28, 18, 12, 8; (**b**) targeted therapy 68, 60, 40, 32, 20, 10; no targeted therapy 86, 51, 29, 16, 8, 5

Immunotherapy was not associated with overall survival (median 24.8 vs. 15.7 months; 12-month overall survival 72.8% vs. 58.1%; HR 0.80, 95% CI 0.49–1.29; *p*=0.355), and no timing window reached significance (Table 2; Fig. 1a).

At 1 year, local, distant intracranial and systemic progression did not differ with immunotherapy or its timing (Table 2); a trend toward lower distant intracranial progression with immunotherapy within 4 weeks before SRS (29.6% vs. 50.7%; *p* = 0.062) did not persist after correction (q = 0.53).

**Table 2:** Survival outcomes and disease progression by immunotherapy exposure and timing (patient level)

	Never received IO (*n* = 67)	IO, any time (*n* = 88)	Before SRS (*n* = 54)	4 weeks before SRS (*n* = 27)	Concurrent (*n* = 25)	After SRS (*n* = 63)	4 weeks after SRS (*n* = 48)
**Overall survival**							
Deaths (n)	31	35	19	8	8	24	20
Median, months (95% CI)	15.7 (0.9–30.6)	24.8 (17.1–32.4)	25.6 (NE)	NR	NR	24.8 (14.8–34.8)	24.8 (16.6–32.9)
12-month, % (95% CI)	58.1 (45.4–70.8)	72.8 (62.6–83.0)	74.2 (61.3–87.1)	72.7 (53.5–91.9)	74.2 (54.2–94.2)	74.5 (62.7–86.3)	74.5 (61.4–87.6)
HR (95% CI)	1	0.80 (0.49–1.29)	0.69 (0.39–1.23)	0.57 (0.26–1.24)	0.59 (0.27–1.29)	0.71 (0.42–1.21)	0.75 (0.43–1.31)
*p (q)*		*0.355*	*0.207 (0.53)*	*0.151 (0.53)*	*0.185 (0.53)*	*0.210 (0.53)*	*0.307 (0.62)*
**1-year progression**,** n (%)**							
Local	30 (44.8)	33 (37.5)	21 (38.9)	10 (37.0)	9 (36.0)	23 (36.5)	19 (39.6)
*p (q)*		*0.361*	*0.514 (0.69)*	*0.492 (0.69)*	*0.449 (0.69)*	*0.338 (0.62)*	*0.579 (0.70)*
Distant intracranial	34 (50.7)	38 (43.2)	20 (37.0)	8 (29.6)	9 (36.0)	29 (46.0)	20 (41.7)
*p* (q)		0.350	0.132 (0.53)	0.062 (0.53)	0.207 (0.53)	0.591 (0.70)	0.336 (0.62)
Systemic	40 (59.7)	59 (67.0)	32 (59.3)	15 (55.6)	14 (56.0)	45 (71.4)	32 (66.7)
*p (q)*		*0.346*	*0.961 (0.96)*	*0.712 (0.79)*	*0.748 (0.79)*	*0.160 (0.53)*	*0.446 (0.69)*

***Notes***: *Abbreviations*: CI = confidence interval; HR = hazard ratio; IO = immunotherapy; NE = not estimable; NR = not reached; OS = overall survival; SRS = stereotactic radiosurgery

Data are presented as n (%) unless otherwise specified. Each subgroup was compared with patients who never received immunotherapy; timing subgroups overlap (Fig. S1). Twelve-month OS rates are Kaplan–Meier estimates. p, log-rank test (OS) or chi-square test (progression); q, Benjamini–Hochberg false discovery rate–adjusted p

Patients who received immunotherapy were more often male and smokers and more often had ECOG 0–1 (70.4% vs. 53.2% of those with documented ECOG; *p* = 0.035) (Table S3). After adjustment for age, sex, ECOG performance status, primary tumor site and number of treated metastases (*n* = 142), immunotherapy was not associated with overall survival (HR 0.79, 95% CI 0.46–1.35; *p* = 0.383), 1-year local (aOR 0.61, 95% CI 0.29–1.31), distant intracranial (aOR 0.91, 95% CI 0.41–2.00) or systemic progression (aOR 1.40, 95% CI 0.68–2.92), or toxicity (aOR 0.87, 95% CI 0.41–1.83). ECOG ≥ 2 (HR 2.15, 95% CI 1.28–3.64) and male sex (HR 1.89, 95% CI 1.09–3.29) were associated with shorter survival, and a higher number of treated metastases with local (aOR 1.15 per metastasis, 95% CI 1.02–1.30) and distant intracranial progression (aOR 1.45, 95% CI 1.24–1.71).

In an exploratory analysis of patients with NSCLC (*n* = 67; 43 with immunotherapy), immunotherapy was not associated with overall survival (1-year 74.3% vs. 71.0%; *p* = 0.863), local (32.6% vs. 29.2%; *p* = 0.774) or distant intracranial progression (48.8% vs. 45.8%; *p* = 0.813).

#### Targeted therapy

Targeted therapy (*n* = 68; 33 before and 55 after SRS; drug classes in Table S1) was associated with longer overall survival (median 30.4 vs. 14.6 months; HR 0.46, 95% CI 0.28–0.76; *p* = 0.002), both before (HR 0.50, 95% CI 0.26–0.98; *p* = 0.038) and after SRS (HR 0.44, 95% CI 0.26–0.76; *p* = 0.002), and after adjustment (HR 0.45, 95% CI 0.26–0.79; *p* = 0.005) (Fig. 1b; Table S2). It was not associated with 1-year local, distant intracranial or systemic progression.

#### Chemotherapy

Chemotherapy (*n* = 111; 87 before and 81 after SRS) was not significantly associated with overall survival (median 19.2 vs. 30.4 months; *p* = 0.135); post-SRS chemotherapy showed a trend toward shorter survival (HR 1.73, 95% CI 0.94–3.16; *p* = 0.074) (Table S2).

Post-SRS chemotherapy was associated with higher 1-year distant intracranial progression (56.8% vs. 34.9%; OR 2.45, 95% CI 1.14–5.28; *p* = 0.020), with no significant association with local or systemic progression (Table S2).

#### Radiotherapy and metastasis parameters

Patients with 1-year local or distant intracranial progression had more treated metastases (median 3 vs. 2, *p* = 0.004; and 4 vs. 1, *p* < 0.001). The number of metastases was not associated with survival, and neither CITV nor FSRT for at least one metastasis was associated with any outcome (all *p* > 0.05).

#### Anatomical location of metastases

**Fig. 2:**
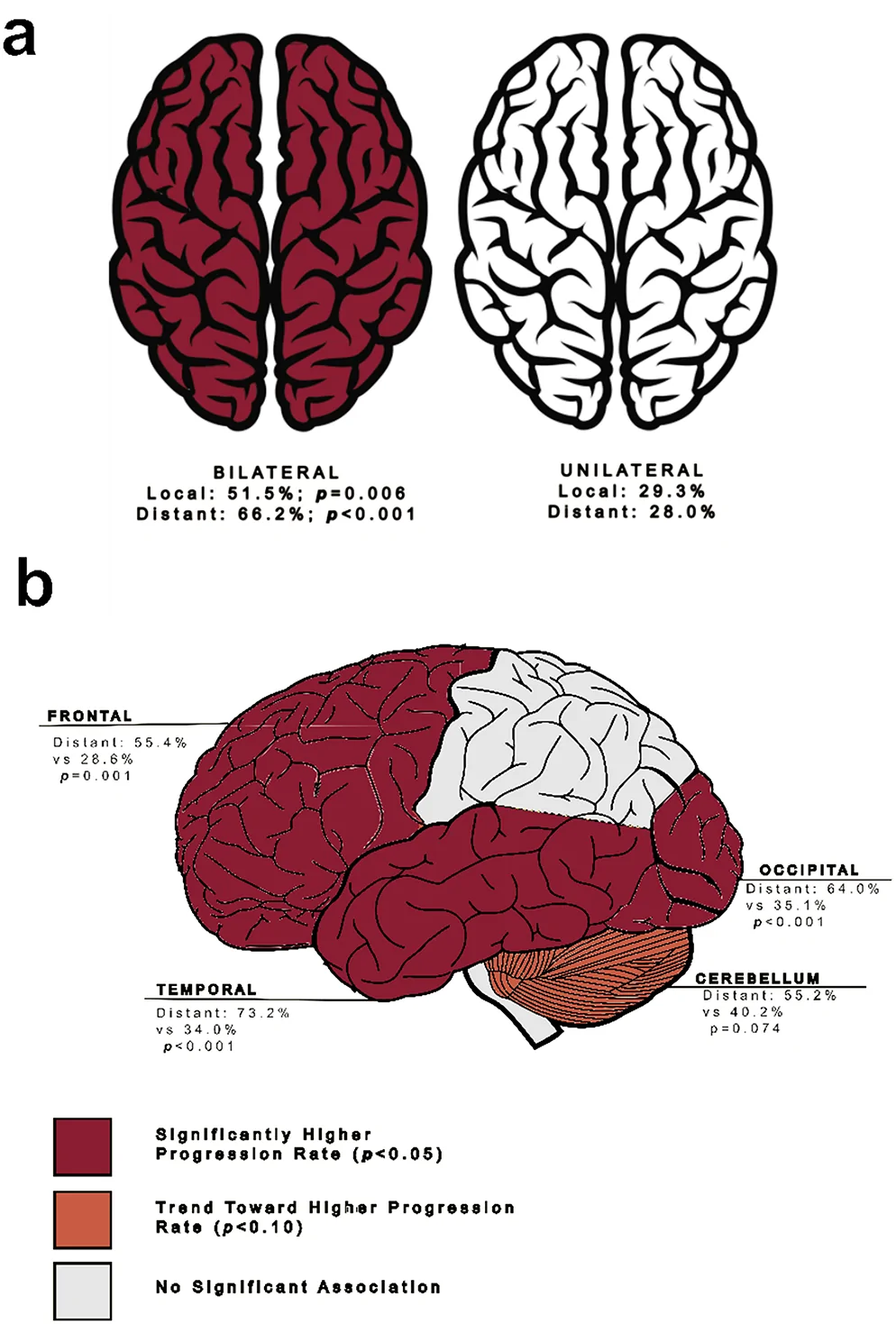
Association of metastasis laterality (**a**) and anatomical location (**b**) with 1-year intracranial progression. (**a**) 1-year local and distant intracranial progression in patients with bilateral versus unilateral metastases. (**b**) 1-year distant intracranial progression in patients with versus without involvement of each site. Colors indicate statistical significance; p, chi-square test. Patients could have metastases in more than one location

Bilateral disease (*n* = 68) was associated with higher 1-year local (51.5% vs. 29.3%; *p* = 0.006) and distant intracranial progression (66.2% vs. 28.0%; *p* < 0.001) (Fig. 2a).

Distant intracranial progression was also more frequent with temporal (73.2% vs. 34.0%; *p* < 0.001), occipital (64.0% vs. 35.1%; *p* < 0.001) and frontal involvement (55.4% vs. 28.6%; *p* = 0.001), with a trend for cerebellar involvement (55.2% vs. 40.2%; *p* = 0.074) (Fig. 2b); no location was associated with local or systemic progression or with overall survival.

### Toxicity

Neither toxicity nor radionecrosis was associated with immunotherapy or chemotherapy, overall or by timing (Table 3).

**Table 3:** Toxicity and radionecrosis by systemic therapy (patient level)

PARAMETERS	ANY-GRADE TOXICITY	*p*-value	RADIONECROSIS	*p*-value
IMMUNOTHERAPY				
Never received IO (*n* = 67)	25 (37.3%)	Reference group	3 (4.5%)	Reference group
IO, at any time (*n* = 88)	26 (29.9%) ᵃ	0.332	9 (10.2%)	0.234
Prior to SRS (*n* = 54)	15 (28.3%) ᵃ	0.298	7 (13.0%)	0.108
Within 4 weeks prior to SRS (*n* = 27)	6 (22.2%)	0.159	1 (3.7%)	1.00
Concurrent (*n* = 25)	5 (20.0%)	0.115	1 (4.0%)	1.00
After SRS (*n* = 63)	18 (28.6%)	0.290	5 (7.9%)	0.483
Within 4 weeks after SRS (*n* = 48)	14 (29.2%)	0.363	4 (8.3%)	0.449
CHEMOTHERAPY ᵇ				
Never received (*n* = 43)	13 (30.2%)	Reference group	2 (4.7%)	Reference group
Chemotherapy, at any time (*n* = 111)	38 (34.5%) ᵃ	0.611	10 (9.0%)	0.512
Prior to SRS (*n* = 87)	34 (39.5%) ᵃ	0.301	9 (10.3%)	0.336
After SRS (*n* = 81)	26 (32.1%)	0.831	6 (7.4%)	0.713
TARGETED THERAPY ᵇ				
Never received (*n* = 86)	31 (36.5%) ᵃ	Reference group	3 (3.5%)	Reference group
Targeted therapy, at any time (*n* = 68)	20 (29.4%)	0.357	9 (13.2%)	0.034 ᶜ
Prior to SRS (*n* = 33)	8 (24.2%)	0.205	3 (9.1%)	0.346
After SRS (*n* = 55)	16 (29.1%)	0.367	7 (12.7%)	0.048 ᶜ

Notes: Abbreviations: CTCAE = Common Terminology Criteria for Adverse Events; IO = immunotherapy; SRS = stereotactic radiosurgery

Data are presented as n (%). Toxicity was graded according to CTCAE v5.0 (highest grade per patient). Each group was compared with patients who never received the corresponding therapy (reference group); timing subgroups overlap (Fig. S1). p values: chi-square test (toxicity) and Fisher’s exact test (radionecrosis)

ᵃ Toxicity grade not documented for one patient

ᵇ Exploratory analyses

ᶜ No longer significant after accounting for bevacizumab started after the diagnosis of radionecrosis (see Results)

Radionecrosis was more frequent with targeted therapy (13.2% vs. 3.5%; *p* = 0.034), including after SRS (12.7% vs. 3.5%; *p* = 0.048), but not before SRS (9.1%; *p* = 0.346); any-grade toxicity did not differ (Table 3).

In two patients, bevacizumab was started after the diagnosis of radionecrosis, as its treatment; when they were classified as unexposed, the associations were no longer significant (any targeted therapy, 10.6% vs. 5.7%; *p* = 0.364; post-SRS, 9.4% vs. 5.7%; *p* = 0.502).

## Discussion

In this single-institution real-world cohort of 155 patients treated with SRS, immunotherapy was not associated with overall survival, intracranial or systemic progression, or toxicity in unadjusted or adjusted analyses, and no immunotherapy timing window remained significant after correction for multiple comparisons.

These findings differ from reports that close temporal proximity between SRS and immune checkpoint blockade improves outcomes. In an individual patient data meta-analysis, concurrent administration was associated with higher 1-year overall survival (64.6% vs. 51.6%) and better regional brain control, although the authors considered these results hypothesis-generating [16]; similar associations have been reported in large cohorts [10, 15, 17]. In our cohort, hazard ratios for the windows closest to SRS (0.57–0.59) pointed in the same direction, but subgroups of 25–27 patients were underpowered, and tumor heterogeneity may have diluted an effect, although results were similar in the NSCLC subgroup.

Immunotherapy, regardless of timing, was not associated with toxicity or radionecrosis. Our findings are consistent with series in which lesion size and dose, rather than timing, predicted radionecrosis [15, 17]. Nevertheless, the literature remains mixed, likely reflecting heterogeneous definitions of toxicity and concurrency [11, 12, 24–26], and dual checkpoint blockade has been linked to increased symptomatic radionecrosis [27].

Post-SRS chemotherapy showed a trend toward shorter survival and was associated with more distant intracranial progression, most plausibly reflecting confounding by indication, as chemotherapy after SRS may have been started for progressive or treatment-resistant disease; systemic therapy sequencing is closely intertwined with disease trajectory [28].

Targeted therapy was associated with longer overall survival, both before and after SRS and after adjustment for performance status. This should be interpreted cautiously, as targeted agents are given to patients with actionable alterations, who form prognostically favorable subgroups [13, 19], and molecular data were unavailable. Radionecrosis was more frequent with targeted therapy, including after SRS, consistent with reports for BRAF inhibitors [29], tyrosine kinase inhibitors [30] and other agents given close to SRS [26]. However, this association rested on few events and was no longer significant after accounting for two patients in whom bevacizumab, itself a treatment for radionecrosis, was started after the diagnosis. Given the heterogeneity of agents, these findings are hypothesis-generating and do not define an optimal interval between SRS and targeted therapy.

A higher number of treated metastases was independently associated with local and distant intracranial progression, consistent with intracranial disease burden as a predictor of new lesions [31]; bilateral disease showed a similar association [3, 11, 21]. Distant progression was also more frequent with temporal, occipital and frontal involvement; because patients with more metastases more often have several lobes involved, these associations probably reflect disease burden rather than lobe-specific biology.

This study has several limitations. The retrospective design introduces selection bias and confounding by indication: sequencing was decided individually in multidisciplinary discussion, for example with upfront SRS for large symptomatic lesions, and timing categories and treatment classes overlapped. Post-SRS exposures are susceptible to immortal-time bias, which could not be addressed because start dates of post-SRS therapies were not recorded. Extracranial disease status, molecular profile, treatment line and RPA class were unavailable, and performance status was recorded only as ECOG. Histological subtypes were documented only for lung primaries, and relatively radioresistant tumors such as melanoma, renal and gastrointestinal primaries may differ in local control and radionecrosis risk. Histology-specific analyses were limited to an exploratory NSCLC subgroup, in which patients without immunotherapy likely included those with driver mutations treated with targeted therapy, and several subgroups were small. Toxicity was graded retrospectively, which may underestimate low-grade events; radionecrosis was diagnosed on conventional MRI without routine perfusion imaging or histology, which limits its distinction from tumor progression [32], and imaging intervals varied. Treatment plans followed published normal-brain dose–volume guidance (e.g., V12 Gy below approximately 5–10 cm³ for single-fraction SRS) [33], but these parameters were not systematically extracted for this analysis. Finally, WBRT was not recorded and may have contributed to radionecrosis and intracranial control, and near-universal corticosteroid use precluded assessment of their effect.

Larger, histologically homogeneous prospective cohorts integrating molecular data are needed to define the optimal sequencing of systemic therapy and SRS.

## Conclusion

In this single-institution real-world cohort, immunotherapy and its timing relative to SRS were not associated with survival, intracranial control or toxicity. Targeted therapy was associated with longer survival in exploratory analyses, whereas its association with radionecrosis was not robust, and intracranial disease burden was the main determinant of intracranial progression. Prospective, histology-specific studies are needed to define the optimal sequencing of systemic therapy and SRS.

## Supplementary Information

Below is the link to the electronic supplementary material.

Supplementary Material 1

## Data Availability

All data supporting the findings of this study are available within the manuscript and its supplementary information files.
