# Supplementary material for: Timing of systemic therapy relative to stereotactic radiosurgery for brain metastases: a real-world cohort study
Source: J Neurooncol. 2026 Nov 3;180(2):36. doi: 10.1007/s11060-026-05857-0 (PMC13731688; doi:10.1007/s11060-026-05857-0)
Supplement: Supplementary file 1 [file 11060_2026_5857_MOESM1_ESM.docx]

**Online Resource 1**

**Article name:** "Timing of Systemic Therapy Relative to Stereotactic Radiosurgery for Brain Metastases: A Real-World Cohort Study"

**Author names:** Mijal Ptascinsky, Antoni Skripai, Yair Hillman, Marc Wygoda, Aron Popovtzer, Tal Falick Michaeli

**Corresponding author:** Tal Falick Michaeli, [tal.michaeli@mail.huji.ac.il](mailto:tal.michaeli@mail.huji.ac.il)

1. Fig. S1	Schematic representation of systemic therapy timing categories
2. Table S1 Targeted therapy by drug class and primary tumor site
3. Table S2 Survival outcomes and disease progression by chemotherapy and targeted therapy exposure
4. Table S3 Baseline characteristics of patients who did and did not receive immunotherapy
a. **Timing categories (immunotherapy)**

b.**Example patients**

**Fig S1. Schematic representation of systemic therapy timing categories.** (A) Timing categories defined relative to the first SRS course (day 0). Concurrent and 4-week categories were defined for immunotherapy only; for chemotherapy and targeted therapy, only pre-SRS and post-SRS administration was assessed. (B) Example patients illustrating that categories are not mutually exclusive; a patient receiving immunotherapy before, around and after SRS contributes to all five categories.

**Table S1. Targeted therapy by drug class and primary tumor site (n=68)**

| **Primary site** | **Kinase inhibitor** | **Monoclonal antibody** | **ADC** | **Other** | **More than 1 class** |
| --- | --- | --- | --- | --- | --- |
| Lung (n=28) | 3 (10.7%) | 20 (71.4%) | 0 | 0 | 5 (17.9%) |
| Melanoma (n=6) | 4 (66.7%) | 0 | 0 | 1 (16.7%) | 1 (16.7%) |
| Breast (n=13) | 2 (15.4%) | 2 (15.4%) | 2 (15.4%) | 0 | 7 (53.8%) |
| GI (n=9) | 0 | 8 (88.9%) | 1 (11.1%) | 0 | 0 |
| Gyn (n=3) | 0 | 1 (33.3%) | 0 | 0 | 2 (66.7%) |
| Kidney (n=3) | 2 (66.7%) | 1 (33.3%) | 0 | 0 | 0 |
| Other (n=5) | 1 (20.0%) | 1 (20.0%) | 2 (40.0%) | 0 | 1 (20.0%) |
| ND (n=1) | 0 | 1 (100.0%) | 0 | 0 | 0 |
| Total (n=68) | 12 (17.6%) | 34 (50.0%) | 5 (7.4%) | 1 (1.5%) | 16 (23.5%) |

***Table S1. notes:***
*Abbreviations*: GI = gastrointestinal; Gyn = gynecologic; ND = not documented; ADC = antibody–drug conjugates**.**
Values are n (%), with percentages calculated within each primary tumor site.
Kinase inhibitors: osimertinib, alectinib, lorlatinib, dabrafenib, trametinib, vemurafenib, encorafenib, binimetinib, cobimetinib, cabozantinib, lenvatinib, pazopanib, regorafenib, sorafenib, palbociclib.
Monoclonal antibodies: bevacizumab, trastuzumab, pertuzumab, cetuximab, amivantamab, and rituximab.
Antibody–drug conjugates: trastuzumab deruxtecan, trastuzumab emtansine, sacituzumab govitecan, enfortumab vedotin, mirvetuximab soravtansine, zilovertamab vedotin, ABBV-706. Other: investigational agent (ARRY-440).
Bevacizumab was received by 35 patients (51.5%).

**Table S2. Survival outcomes and disease progression by chemotherapy and targeted therapy exposure (exploratory analyses)**

| **Parameters** | **Median OS, months (95% CI)** | **12-months OS, % (95% CI)** | **HR (95% CI)** | **1-year local progression** | **1-year distant intracranial progression** | **1-year systemic progression** |
| --- | --- | --- | --- | --- | --- | --- |
| Chemotherapy |  |  |  |  |  |  |
| Never received (n=43) | 30.4 (18.8-41.9) | 74.5 (60.0-89.0) | Reference group | 16 (37.2%) | 15 (34.9%) | 28 (65.1%) |
| Chemotherapy, at any time (n=111) | 19.2 (12.3-26.0) | 63.2 (53.6-72.8) | 1.56 (0.87-2.83) *p=0.135* | 47 (42.3%) *p=0.561* | 56 (50.5%) *p=0.082* | 71 (64.0%) *p=0.893* |
| Prior to SRS (n=87) | 21.7 (NE) | 62.2 (51.2-73.2) | 1.45 (0.78-2.68) *p=0.234* | 40 (46.0%) *p=0.342* | 44 (50.6%) *p=0.091* | 55 (63.2%) *p=0.832* |
| After SRS (n=81) | 18.8 (14.2-23.4) | 61.0 (49.6-72.4) | 1.73 (0.94-3.16) *p=0.074* | 32 (39.5%) *p=0.803* | 46 (56.8%) ***p****=0.020* | 60 (74.1%) *p=0.296* |
| TARGETED THERAPY |  |  |  |  |  |  |
| Never received (n=86) | 14.6 (11.3-17.9) | 58.2 (46.6-69.8) | Reference group | 31 (36.0%) | 38 (44.2%) | 53 (61.6%) |
| Targeted therapy, at any time (n=68) | 30.4 (NE) | 75.7 (64.9-86.5) | 0.46 (0.28-0.76) ***p****=0.002* | 32 (47.1%) *p=0.168* | 33 (48.5%) *p=0.591* | 46 (67.6%) *p=0.439* |
| Prior to SRS (n=33) | NR | 69.1 (52.0-86.2) | 0.50 (0.26-0.98) ***p****=0.038* | 14 (42.4%) *p=0.521* | 12 (36.4%) *p=0.439* | 22 (66.7%) *p=0.610* |
| After SRS (n=55) | 30.4 (NE) | 78.6 (67.2-90.0) | 0.44 (0.26-0.76) ***p****=0.002* | 27 (49.1%) *p=0.125* | 26 (47.3%) *p=0.72* | 37 (67.3%) *p=0.496* |

***Table S2. Notes:***  *Abbreviations***:** CI = confidence interval; CT = chemotherapy; HR = hazard ratio; NE = not estimable; NR = not reached; OS = overall survival; SRS = stereotactic radiosurgery; TT = targeted therapy.
Data are presented as n (%) unless otherwise specified. Each subgroup was compared with patients who never received the corresponding therapy; timing subgroups overlap (Fig.S1). Twelve-month OS rates are Kaplan-Meier estimates. HR, unadjusted hazard ratio (Cox regression); p, log-rank test (OS) or chi-square test (progression). Post-SRS exposures are susceptible to immortal-time bias.

**Table S3. Baseline characteristics of patients who did and did not receive immunotherapy**

| **p-value** | **Immunotherapy (n=88)** | **No immunotherapy (n=67)** | **PARAMETERS** |
| --- | --- | --- | --- |
|  |  |  | DEMOGRAPHIC |
| 0.397 | 67 (36-93) | 66 (16-85) | Age (years) |
| **0.012** |  |  | Sex |
|  | 36 (40.9%) | 41 (61.2%) | Female |
|  | 52 (59.1%) | 26 (38.8%) | Male |
| **0.005** | 58 (65.9%) | 29 (43.3%) | Current or former smoker |
|  |  |  | BASELINE CLINICAL CHARACTERISTICS |
| 0.933 | 9 (6-13) | 9 (6-15) | Charlson comorbidity index |
| **0.035 ᵃ** |  |  | ECOG performance status |
|  | 57 (64.8%) | 33 (49.3%) | 0-1 |
|  | 24 (27.3%) | 29 (43.3%) | ≥2 |
|  | 7 (8.0%) | 5 (7.5%) | Not documented |
| 1.00 | 84 (95.5%) | 64 (95.5%) | Corticosteroids on the day of SRS |
| **<.001 ᵇ** |  |  | PRIMARY TUMOR SITE |
|  | 45 (51.1%) | 32 (47.8%) | Lung |
|  | 18 (20.5%) | 1 (1.5%) | Melanoma |
|  | 2 (2.3%) | 16 (23.9%) | Breast |
|  | 1 (1.1%) | 10 (14.9%) | Gastrointestinal |
|  | 5 (5.7%) | 4 (6.0%) | Gynecological |
|  | 8 (9.1%) | 0 | Kidney |
|  | 8 (9.1%) | 4 (6.0%) | Other |
|  | 1 (1.1%) | 0 | Not documented |
|  |  |  | INTRACRANIAL DISEASE |
| 0.176 | 2 (1-15) | 2 (1-27) | Number of treated metastases per patient |
| 0.747 | 1.38 (0.02-34.30) | 1.48 (0.03-28.40) | Cumulative intracranial tumor volume (cm^3^) |
| 0.756 | 31 (35.2%) | 22 (32.8%) | Synchronous |
| 0.860 | 38 (43.2%) | 30 (44.8%) | Bilateral metastasis |
| 0.147 | 39 (44.3%) | 22 (32.8%) | FSRT for ≥1 metastasis |
| 0.181 | 0 | 2 (3.0%) | Leptomeningeal spread |
|  |  |  | OTHER SYSTEMIC TREATMENTS |
| **0.049** | 58 (65.9%) | 53 (79.1%) | Chemotherapy ^c^ |
| 0.349 | 36 (40.9%) | 32 (47.8%) | Targeted therapy |
| **0.013** | 0 | 5 (7.5%) | Hormonal therapy |

***Table S3. Notes:***  *Abbreviations***:** ECOG = Eastern Cooperative Oncology Group; FSRT = Fractionated Stereotactic Radiotherapy; SRS = Stereotactic Radiosurgery.
Data are presented as N (%) or median (range). p values: chi-square or Fisher’s exact test (categorical variables) and Mann-Whitney U test (continuous variables).

ᵃ ECOG 0-1 vs ≥2, among patients with documented ECOG.
ᵇ Overall comparison across primary tumor sites.
ᶜ Not documented for one patient without immunotherapy.
